# Analyzing the Satellite-Induced Code Bias Variation Characteristics for the BDS-3 Via a 40 m Dish Antenna

**DOI:** 10.3390/s20051339

**Published:** 2020-02-29

**Authors:** Ju Hong, Rui Tu, Rui Zhang, Lihong Fan, Pengfei Zhang, Junqiang Han, Xiaochun Lu

**Affiliations:** 1 National Time Service Center, Chinese Academy of Sciences, Xi’an 710600, China; hongju@ntsc.ac.cn (J.H.); zhangrui@ntsc.ac.cn (R.Z.); fanlihong@ntsc.ac.cn (L.F.); zhangpengfei@ntsc.ac.cn (P.Z.); hanjunqiang@ntsc.ac.cn (J.H.); luxc@ntsc.ac.cn (X.L.); 2 University of Chinese Academy of Sciences, Beijing 100049, China; 3 Key Laboratory of Precision Navigation and Timing Technology, Chinese Academy of Sciences, Xi’an 710600, China

**Keywords:** third-generation BDS, 40 m dish antenna, satellite-induced code bias variation, characteristics analysis

## Abstract

The satellite-induced code bias variation of geostationary satellite orbit satellites and medium earth orbit satellites of the second-generation BeiDou Navigation Satellite System (BDS-2) exceeds 1 m, which severely affects the accuracy and stability of the ambiguity resolution and high-precision positioning. With the development of the third-generation BDS (BDS-3) with a new system design and new technology, analysis of the satellite-induced code variation characteristics of BDS-3 has become increasingly important. At present, many scholars have explored the satellite-induced code bias of BDS-3, but most of them focus on BDS-3 experimental satellites via normal geodetic antenna. Compared to normal geodetic antenna, the 40-m dish antenna from the National Time Service Center can accurately detect satellite-induced code variations with low noise and high gain. Thus, observational data from fifteen BDS-3 medium earth orbit satellites are collected with the B1I/B2b/B3I/B1C/B2a frequency bands on the day of year (DOY) 199–206 in 2019, the PRN numbers of which are C19/C20/C21/C22/C23/C24/C25/C26/C27/C28/C30/C32/C33 /C35/C37, via the 40 m dish antenna to analyze the code bias variation characteristics. The results show that the obvious satellite-induced elevation‑dependent code bias variations exist in the B1I/B2b/B3I/B1C/B2a frequency bands of C28, compared with other satellites. Similarly, the multipath (MP) combination of B3I has an obvious elevation‑dependent variation within a range of 0.1 m for C21/C24/C27/C28/C37 and elevation‑dependent variation of the B2a and B2b frequency bands also exists in most satellites with a range of 0.1 m. However, the MP combination values of some satellites are asymmetric with respect to elevation, which is different from BDS-2 satellites and especially obvious for BDS-3 satellites B1I and BIC frequency bands with elevation‑dependent variations of 0.2 m, indicating that the code bias variation is not uniquely related to elevation, especially for the B1I/BIC frequency bands. What’s more, the satellite-induced code bias variation of the BDS-3 satellites is greatly reduced compared with that of the BDS-2 satellites. In addition, the similar code bias variation appears at the Xia1 station with a normal geodetic antenna of B1I/B1C/B3I/B2a/B2b of C21, B3I/B2a/B2b of C24 and B2b of C28 among B1I/B1C/B3I/B2a/B2b of C21/C24/C27/C28/C37. The influence of the BDS-3 satellite-induced elevation‑dependent code bias on precision positioning and ambiguity fixing is worth further study using different antennas or receivers.

## 1. Introduction

The BeiDou Navigation Satellite System (BDS) is a global satellite navigation system developed by China. The successful launch of the first group of networking satellites on 5 November 2017 marked the start of the third-generation BDS (known as BDS-3) entering the global construction stage. As of December 2019, all three inclined geostationary satellite orbit satellites (IGSO) and twenty-four medium earth orbit (MEO) satellites of BDS-3 have been easily launched (http://www.beido.u.gov.cn), and the completion of MEO satellite networking represents the completion of the core constellation deployment of BDS-3. Compared to the BDS-2 satellites, the new system signals, B1C and B2a, which are the two major public service signals of BDS-3, are broadcast on the BDS-3 satellites. With the construction of four global navigation satellite systems (GNSS), the compatibility and interoperability between systems has become increasingly important. The B1C signal shares a frequency band (1575.420 MHz) with the global positioning system (GPS) L1 and Galileo E1 civil signals, and the BDS B2a/GPS L5/Galileo E5a signals share the same frequency band (1176.450 MHz), which means that BDS-3 further integrates into the family of GNSS and will also bring significant changes in satellite navigation receiver technology, including improvements to the service performance and reductions in power consumption and the cost of equipment.

To a great extent, the accuracy, reliability, and availability of satellite navigation signals depend on the observation quality of satellite navigation signals in which the code-phase divergences (code bias variations) are an important part of the quality evaluation, which is a prerequisite for ambiguity resolution [1] and high-precision absolute positioning such as precise point positioning (PPP) [2,3]. Hauschild et al. [4] first determined that the code-phase divergences exist based on COMPASS-M1 tracking data. Many scholars have done in-depth research on identifying code-phase divergences in BDS-2 with diverse COMPASS-capable multi-frequency GNSS receivers and various data sources made public, for example Multi-GNSS Experiment (MGEX) [5]. The satellite-induced code bias variations are about 1 m from the horizontal range to the zenith for BDS IGSOs and MEOs, which is related to the elevation, frequency, and orbit type [2,3,6,7,8]. In GNSS data processing, the code bias variation severely affects the accuracy and stability of wide-lane ambiguity that is derived from the Melbourne–Wübbena (MW) combination and finally degrades the precision of BDS PPP ambiguity estimations for BDS-2 satellites [9,10]. Additionally, a very strong systematic effect of about 1 m is found in the height component in single-frequency ionosphere-free combination PPPs [2].

The code variations of the BDS-3 experimental satellites and BDS-3 satellites have already been discussed in previous research with the development of BDS-3 system [11,12,13,14,15,16]. Zhang et al. [13,14] analyzed all signals based on the two IGSO and three MEO experimental satellites measured by a single receiver and multiple receivers, respectively, using normal geodetic antennas; their results show that the satellite-induced code bias for BDS-3 experimental satellites and signals can be negligible for observations observed at different stations’ MW values. Some studies proved that the code bias variation of BDS-3 satellite with normal geodetic antennas is less than that of BDS-2 satellite and there is no apparent satellite-induced code bias on BDS-3 satellites [16,17,18]. The code observations for BDS-3 satellites, however, may be contaminated due to the low gain of normal geodetic antennas and multipath (MP) error from the surroundings. Zhou et al. [15] noted that the observation of 40 m dish antenna from the National Time Service Center of the Chinese Academy of Sciences provides a way to analyze these types of systematic code variations since the MP combination observations got by it accurately detected the satellite-induced code variation without considering MP errors resulting from the station surroundings. Meantime, they used the 40-m dish antenna to gain more insight into the code-carrier divergence characteristics of BDS-3 experimental satellites, identifying the existence of code bias variations in a range of 0.1 m. There are differences between the BDS-3 experimental satellites and the BDS-3 satellites, and the existing research lacks the more accurate research on the satellite-induced code bias variation BDS-3 satellite. In this study, we therefore analyzed MP combination value from the 40 m dish antenna to provide accurate code bias characteristics for BDS-3 satellites.

This paper first provides an overview on the BeiDou system and data sources, then introduces the MP combination formula used to analyze the code bias, and finally analyzes the elevation‑dependent code bias, B1C_data and B1C_pilot code bias, and compares the code bias of observations between the normal geodetic antenna and the 40-m dish antenna.

## 2. Development of the BeiDou System

**Figure 1:**
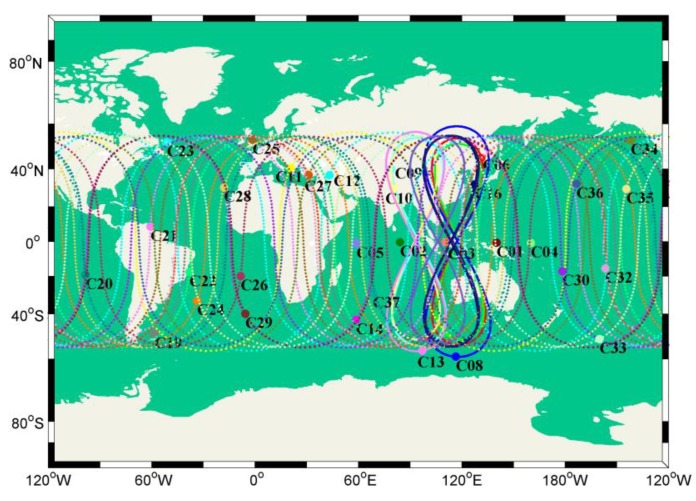
Footprints of BDS-2 and BDS-3 operational satellites.

The BeiDou system follows the principles of step-by-step construction according to a three-step master plan. The BeiDou test system started development in 1994, and then two Geostationary Earth Orbit (GEO) satellites were launched successfully, forming the first generation of the BDS (BDS-1) in 2000 [19]. The active positioning system was adopted to provide positioning, time service, wide area difference, and short message communication services for Chinese users. China started to develop BDS-2 in 2004 and successfully completed the deployment of 14 satellite networks by the end of 2012, which cover the Asia-Pacific region and includes a passive location system [20]. On 5 November, 2017, China officially began to build the BeiDou global satellite navigation system (BDS-3). The BDS-3 system inherits both the active service and passive service, which can provide basic navigation (positioning, speed measurement, time service), global short message communication, and international search and rescue services for global users [21]. Figure 1 shows the ground tracks of BDS-2 and BDS-3 satellites in normal operation. The existing satellites have full navigation service capability in global area. BDS-3 is downward compatible with B1I and B3I signals as well as the addition of new B1C and B2a signals. The frequencies, code rates, and modulation scheme of the BDS-3 signals are summarized in Table 1 [22,23]. In addition to the public service signals in Table 1, there are B1A in Binary Offset Carrier (BOC) (1,1) modulation, B3C Quadrature Phase Shift Keying (QPSK) (10) modulation and B3A in BOC (15,2.5) modulation authorized service signals [22], which are not be described in detail here. The B2 signals are divided into B2a and B2b signals similar to the E5a and E5b bands of Galileo. The B2a signals, with the center frequency at 1176.45 MHz, are transmitted in QPSK (10) modulation. It is worth noting that the B2a of BDS-3 replaces the B2I of BDS-2 with different frequency and modulation modes, while B2b and B2I share the same frequency but have different modulation modes. The B1C signal is divided into two components, B1C_data and B1C_pilot, which are respectively transmitted by the Quadrature Multiplexed Binary Offset Carrier (QMBOC) (6,1) and BOC (1,1) modulation with a center frequency of 1575.42 MHz.

## 3. MP Combinations

MP combination values mainly reflect the MP error and observation noise of satellites and receivers; since the variations in the MP combination values are similar at different stations, it was determined that satellite-induced code bias exists in the BDS-2 satellite. The MP combination is a geometry-free and ionosphere-free combination formed by a one-frequency code and two-frequency phase measurements [24]. Through the linear combination of observations, the MP combination eliminates the influence of the orbit, clock difference, troposphere, and first-order ionosphere, including only the ambiguity, MP error and observation noise of the code and phase combination values, primarily. Compared with the MP error and observation noise of the code, the carrier MP error and observation noise are negligible [25]. During a continuous observation arc in which the ambiguity of a satellite is a constant, if the MP combination value changes, it must be caused by the code bias at the satellite end or the receiver end by the signal reflection. The MP combination formula is as follows [2,26]:

(1) $${\mathrm{MP}}_{i}=P_{i}-\frac{f_{i}^{2}+f_{j}^{2}}{f_{i}^{2}-f_{j}^{2}}L_{i}+\frac{2f_{j}^{2}}{f_{i}^{2}-f_{j}^{2}}L_{j}=M_{P_{i}}+B_{i}+M_{\varphi_{i}},\;$$

where subscripts *i* and *j* denote different signal frequency bands. *P* is the code measurement, *L* is the phase measurement, and *f* is the frequency value. *B* denotes the phase ambiguity bias term and hardware delay biases. $M_{\varphi }$ and $M_{P}$ are the phase and code MP error and observation noise effect, respectively. $M_{P_{i}}$ is the MP combination value of the *i*th frequency band, and *j* can use any frequency band of the same satellite system without influencing the MP combination value. When no carrier phase cycle slips exist, the value may be considered constant. Therefore, after factoring it out with an average term during a continuous observation arc, the residual series are dominated by MP error effects and the tracking noise of the code observations.

## 4. Data Collection

**Figure 2:**
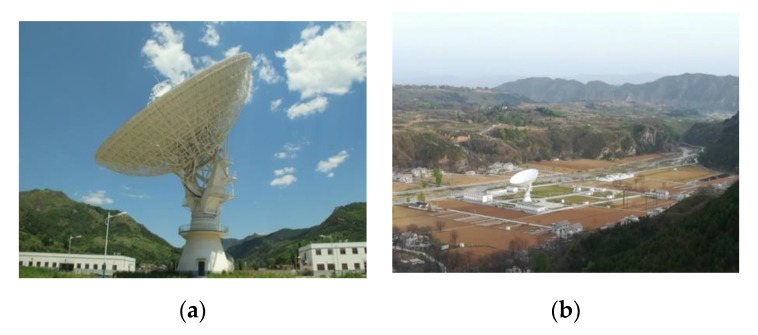
The (**a**) and (**b**) show the 40 m dish antenna and its surrounding environment respectively.

The National Time Service Center of the Chinese Academy of Sciences possesses the GNSS signal quality assessment system of the Haoping 40-meter radio telescope (HRT). The system utilizes a 40 m dish antenna and the receiving/transmission equipment as the core, which can realize the multiple angles, multi-levels, and all-around detection and evaluation of the navigation signal quality in the GNSS by collecting data related to the GNSS satellite space signal quality. The MP combination value is not only related to the satellite signal itself but also to the configuration and environment of the antenna and receiver [16]. The 40-m dish antenna and monitoring receiver II produced by the National University of Defense Technology were used to collect observations to analyze the code bias of the BDS-3 satellites. The 40-m dish antenna, with a high gain for the navigation signal, radiates the signal input power (energy) intensively and ensures a pure electromagnetic environment surrounded by mountains. The observations with low noise are better able to reflect the satellite-induced code bias. In addition, the beam of the 40-m dish antenna is very narrow and highly directional, so only one satellite can be observed at a time. The 40 m dish antenna and its surrounding environment are shown in Figure 2.

In this experiment, the observation data of fifteen BDS-3 MEO satellites are collected with the B1I/B2b/B3I/B1C/B2a frequency bands on the day of year (DOY)199–206 in 2019, the PRN numbers of which are C19/C20/C21/C22/C23/C24/C25/C26/C27/C28/C30/C32/C33/C35/C37 using the NUDT receiver, and the sampling interval is 1 s. Since cycle slips of C35 and C37 B2b observations occur more frequently, so they cannot be used in the experiment. The elevations of most satellites observed are in the range of 10°–85°, contributing to the analysis of the relationship between the MP combination value and elevation. The methods of creating MP combinations with the available frequency bands are shown in Table 2, in which the other frequency band is an arbitrary choice.

## 5. Results and Discussion

**Figure 3:**
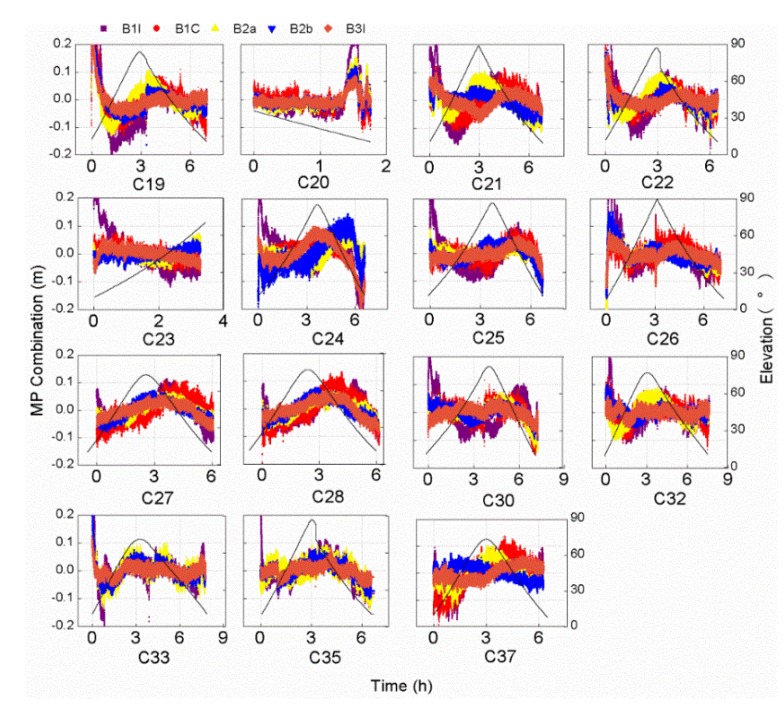
Time series of the MP combinations values and elevations tracked by the 40 m dish antenna for each observed BDS-3 frequency band. The black line represents the elevation, and the violet, red, yellow, blue, and orange points represent the MP combination values of the B1I, B1C, B2a, B2b, and B3I frequency bands, respectively.

**Figure 4:**
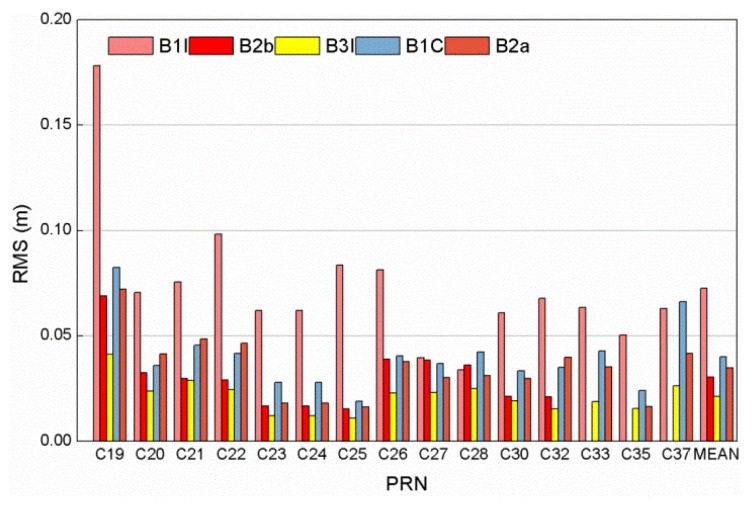
The root mean square (RMS) values of MP combinations tracked by a 40-m dish antenna for each of the BDS-3 satellite frequency bands observed.

This part analyzed and discussed the results. Figure 3 shows the time series of the MP combination values and elevations, in which the black line represents elevations, and the violet, red, yellow, blue, and orange points represent the MP combination values of the B1I, B1C, B2a, B2b, and B3I frequency bands, respectively. In the section, the B1C_pilot signal is used as B1C. The MP combination values of the BDS-3 satellite signals vary a little and concentrated in a range of about 0.2 m, which is smaller compared with BDS-2 satellites. From the relationship between elevation and MP combination values, the MP combination values varies with satellite elevation overall, but the variation is not the same for different satellites. Obviously, MP combination has the largest value when the satellite elevation is less than 30°. The variation of MP combination values is different for available signal bands. In order to further analyze the difference in the code bias of the different frequency bands, Figure 4 shows the root mean square (RMS) values of the MP combination value of all frequency bands in which the last data “MEAN” refers to the RMS values of all satellites.

The RMS value of MP combination values of the frequency bands is noticeably small about 0.05 m but B1I in about 0.1 m, which indicates that MP error resulting from the station surroundings can be ignored and any systematic bias remaining in the MP observations for the experiment can be safely attributed to the satellite or receiver configuration for the 40 m dish antenna [15]. From the overall results, the MP time series of the old signal B3I is the most stable, showing better MP mitigation performance. The MP combination value of the B1I frequency band has the largest RMS value, indicating the weakest anti-MP ability. The B1I signal experiences interference by the B1A signal due to the overlapping of the B1A and BII spectra of the BDS-3 satellite signal, leading to poor signal quality for B1I. The magnitudes of the MP and noise are related to the observed chip rate [27]. The MP mitigation of the B2 signal is better than that of the B1 signal, since the B2 signal has a higher code rate than the B1 signal does; however, the MP time series stability of the B2 signal does not show better performance than that of the B3I signal, although QPSK is more resistant to MP issues than BPSK is.

### 5.1. Elevation‑Dependent Code Bias Analysis

**Figure 5:**
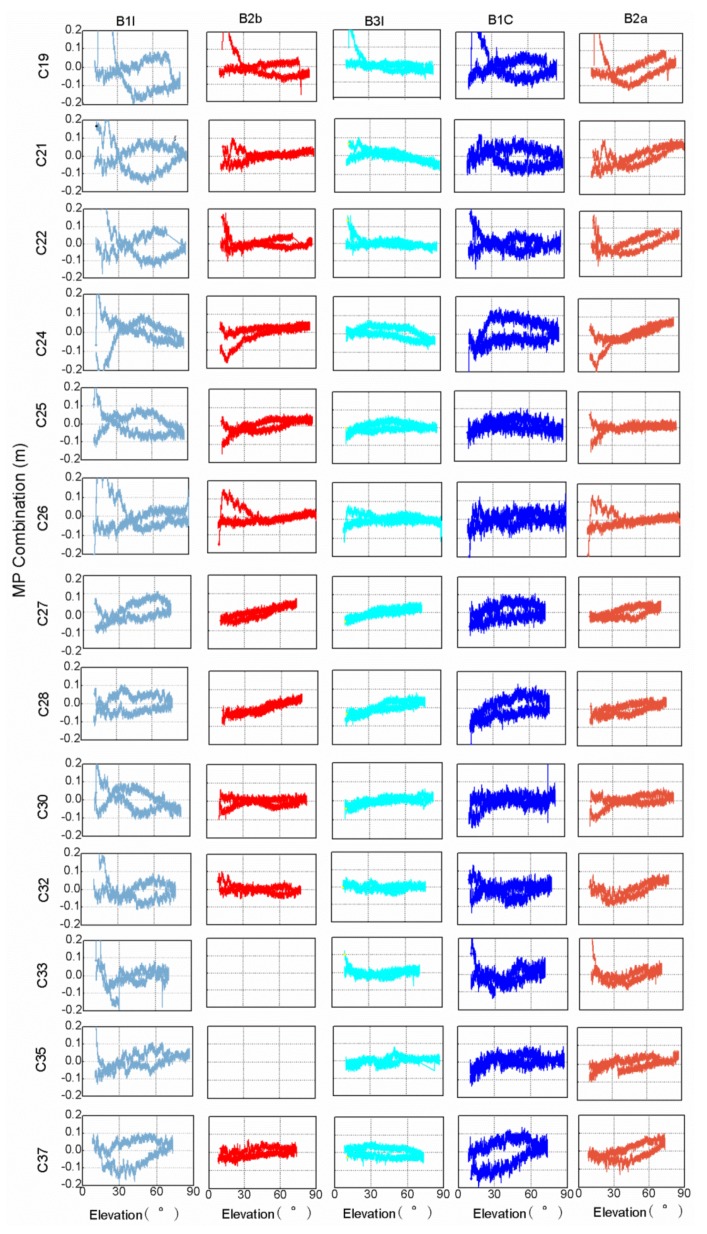
The MP combination time series tracked by a 40 m dish antenna with respect to elevation for each BDS-3 satellite frequency band observed. Different colors represent different frequencies MP combination time series, from left to right, followed by B1I, B2b, B3I, B1C and B2a. Since cycle slips of C35 and C37 B2b observations occur more frequently, the corresponding results are vacant.

The MP combination value of the BDS-2 satellite has a strong correlation with the satellite elevation in earlier findings [2]. It can be seen from Figure 3 that the MP combination values of the BDS-3 satellites vary with elevation. The overall trend of the MP combination time series of the C20 and C23 satellites varies monotonously with elevation, except for the MP combination time series of C20 with elevations below 30°. Other satellites have the highest elevations in the observed arc segment, but the MP combination values of the same elevation are not exactly equal, as is shown in Figure 3. In order to further explore the relationship between the MP combination values and elevations of the BDS-3 satellites, Figure 5 shows the MP combination time series with respect to elevation. There is obvious elevation‑dependent code bias variation for the C28 B1I/B2b/B3I/B1C/B2a frequency bands, compared with those present for other satellites. Similarly, the MP combination of B3I has an obvious elevation‑dependent variation in a range of 0.1 m for C19/C21/C24/C27/C28/C37. The obvious elevation‑dependent variation of the B2a and B2b frequency bands also exists in most satellites with a range of 0.1 m, but the MP combination values of some satellites are asymmetric with respect to elevation, which is especially obvious for the B1I and BIC frequency bands with elevation‑dependent variations of 0.2 m, which indicates that the code bias variation is not uniquely related to elevation, especially for the B1I/BIC frequency bands. This performance also appears in the B1C frequency band for three BDS-3 experimental satellites [15]. The code bias variation may have a certain relationship with elevations and azimuths, which deserves further study with more observations. The code bias variations that are asymmetric with respect to elevation can be considered the satellite-induced code bias variations, since the code bias variations differ from different satellites. What’s more, the satellite-induced code bias variation of the BDS-3 satellites in a range of 0.1-0.2 m is greatly reduced compared with that of the BDS-2 satellites [2,7].

**Figure 6:**
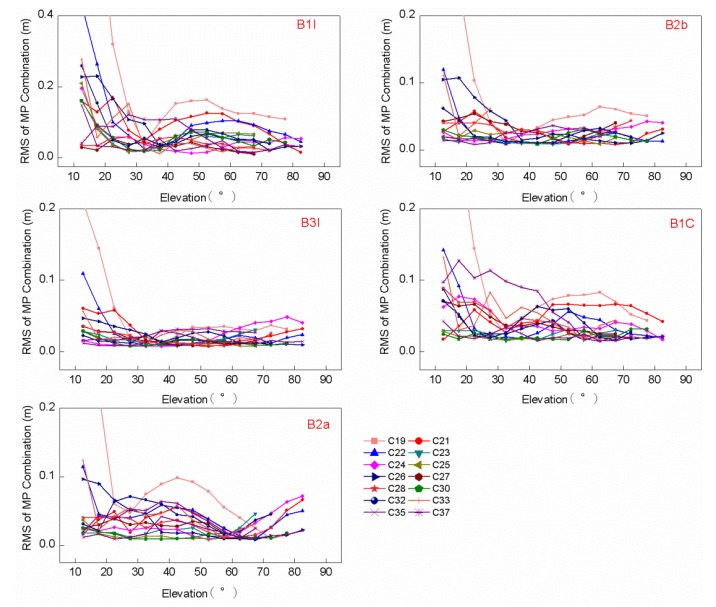
The RMS values of MP combination values tracked by a 40 m dish antenna for elevation ranges of 5° using the BDS-3 satellite frequency band observations of monotonic changes in elevations from 10°–90°.

**Figure 7:**
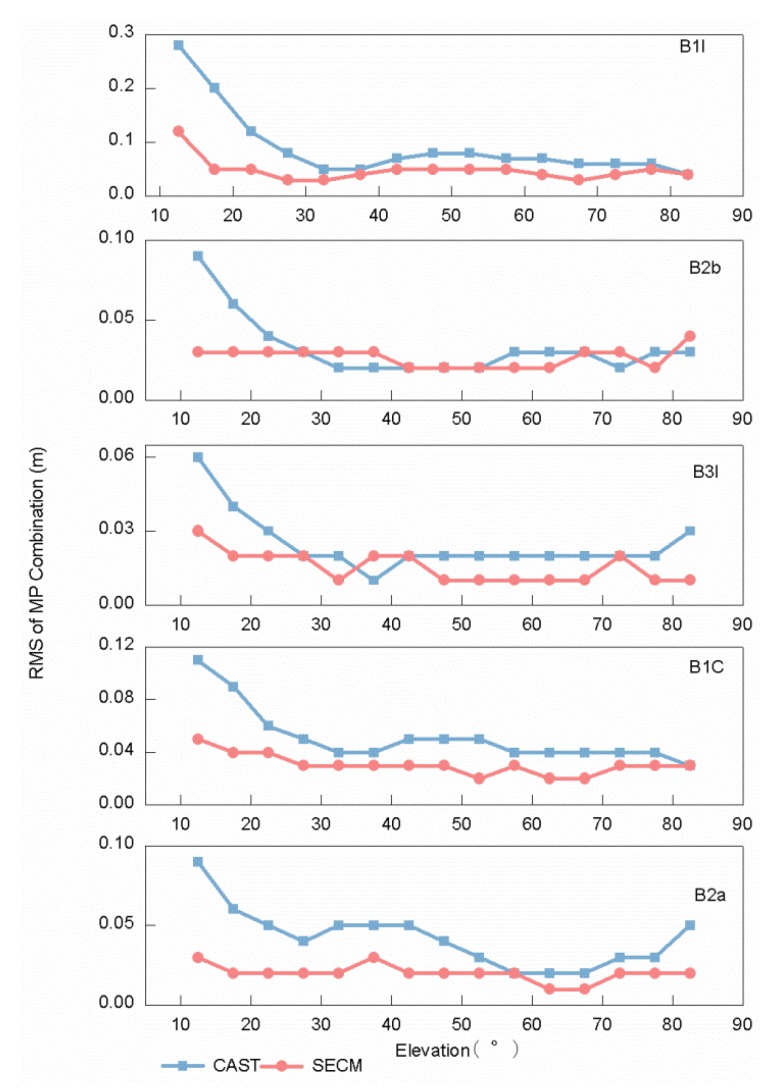
Comparison of RMS values of MP combinations tracked by a 40 m dish antenna between manufacturer A and manufacturer B.

In order to explore the regularity of the same frequency and elevation on different satellites, the RMS values are calculated for every elevation range of 5° for the MP combination values of all frequency bands of monotonic changes in elevations from 10°–90°, which can be seen in Figure 6. As is shown in Figure 5 and Figure 6, the MP combination time series is the most unstable at elevations below 30°, which is most vulnerable to interference from MP errors. This can be considered in the stochastic model of GNSS data processing; however, not all the RMS values and MP combination values decrease with increased elevation all the time. The RMS value of the B1I/B1C frequency bands increases at elevations of 40°–70°; the RMS value of the B2a frequency band increases at elevations of 60°–90°. The BDS-3 satellites are manufactured by two manufacturers, the Shanghai Engineering Center for Microsatellites (SECM) of the China Academy of Science (CAS) and China Academy of Space Technology (CAST) [28]; the RMS values of the MP combination values are shown for both manufacturers in Figure 7. The C19/C21/C22/C23/C24/C32/C33/C37 satellites manufactured by CAST and C25/C26/C27/C28/C30/C35 satellites manufactured by SECM are used to analysis the trends. The code bias of the two manufacturers is different for elevations below 25°, but the difference decreases with the increase in elevation, which indicates that the satellite-induced code variations of the two manufacturers have no obvious difference at elevations greater than 25°.

### 5.2. B1C Signal Components Code Bias Comparison

The new signals B1C and B2a are the two main public service signals of BDS-3 with a wider bandwidth, higher ranging accuracy, and better interoperability. New navigation messages B-CNAV1 and B-CNAV2 are modulated by B1C and B2a, in which the orbit description accuracy and ionosphere correction accuracy are significantly improved compared with BDS-2 with new orbit parameters and global ionosphere model BDGIM based on a spherical harmonic function [21]. The B1 signal with the smallest ionospheric error is the preferred signal for single-frequency users as well as multi-frequency users, so research on the B1C code bias is of great significance for precise positioning. The B1C signal has two signal components, B1C_pilot and B1C_data, which are respectively transmitted by the QMBOC (6,1) and BOC (1,1) modulation mode with a center frequency of 1575.42 MHz. The split-spectrum design of BOC and QMBOC is helpful to improve the ranging accuracy.

**Figure 8:**
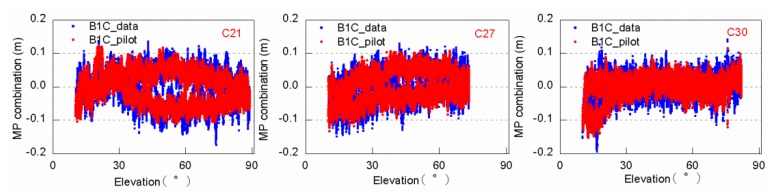
MP combination time series with respect to elevation for C21/C27/C30 B1C_data and B1C_pilot signal tracked by a 40 m dish antenna.

The code bias of the two components may vary due to the different modulation modes, so the RMS values of the two signal components of all satellites are shown in Table 3, in which the last data “MEAN” refers to the mean values of all satellites. Meanwhile, the trends in the MP combination time series are compared in Figure 8. The code bias of the B1C_data is slightly larger than that of the B1C_pilot data, and the MP combination time series have similar variations. The difference is small and can be ignored. Other satellites have demonstrated similar results, which will not be shown herein.

### 5.3. Comparison of the Code Bias with Normal Geodetic Antenna

According to the above analysis, there is a code bias variation of about 0.1–0.2 m in the BDS-3 satellite, which is not uniquely related to the elevation. The observations by the 40-m dish antenna with low noise are better able to reflect the satellite-induced code bias; however, elevation‑dependent code bias may be hidden due to the higher noise and lower gain of the normal geodetic antennas for mass-market. The observations in the same arc section of the Xia1 station with the antenna of RINT-8CH and the receiver of GNSS-GRR from the International GNSS Monitoring & Assessment System (iGMAS) are selected to explore and compare the performance of the code bias for mass users with normal geodetic antennas. The position of the Xia1 station is in Lintong, Xi’an and close to that of the 40-m dish antenna, which ensures the consistency of the elevations and satellite terminal errors within the same observation arc. The systematic bias difference between the Xia1 station and 40-m dish antenna can be attributed to antenna and receiver configurations without MP errors of the antenna and receiver resulting from the surroundings. The iGMAS station only tracks the B1C_pilot signal which was used in the section. The sampling interval of observations by the 40-m dish antenna is changed to be the same as that of the Xia1 station for 30 s.

**Figure 9:**
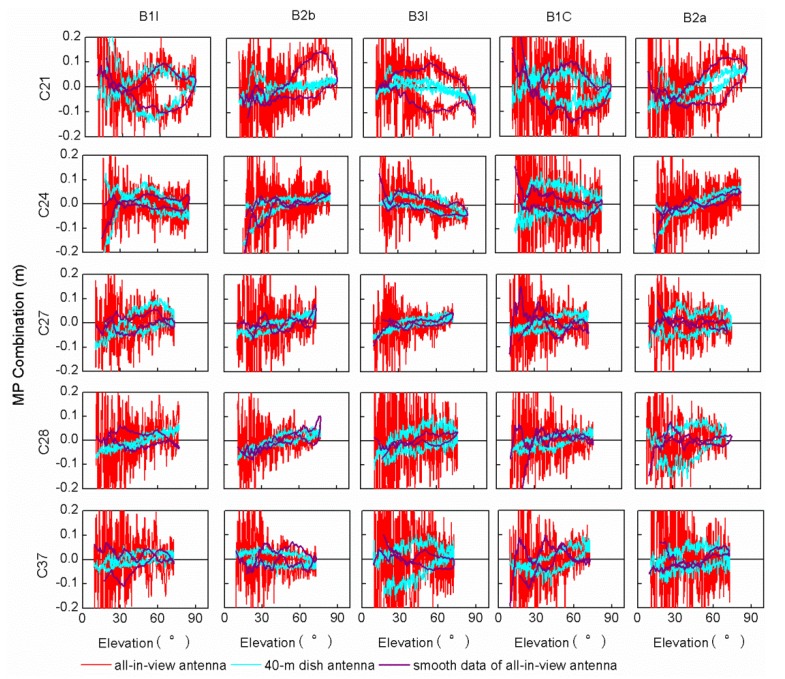
The MP combination time series tracked by two receivers with the 40 m dish antenna and normal geodetic antenna with respect to elevation for the BDS-3 satellites C21/C27/C28/C37 for the available frequency bands.

Table 4 shows the RMS of the MP combination values of each satellite frequency band. The RMS values of the MP combination of Xia1 is about 10 cm for the B1I/B2b/B3I/B2a frequency bands and exceeds 10 cm for the B1C frequency band—larger than that measured by the 40-m antenna. Figure 9 shows the MP combination time series with respect to the elevations of the C21/C24/C27/C28/C37 satellites with obvious elevation‑dependent code bias measured by the 40-m antenna, in which the purple line refers to the smooth data for the Xial station. Among the frequency bands of the C21/C24/C27/C28/C37 satellites in Xia1 station, there still exists an elevation‑dependent code bias of in the B1I/B1C/B3I/B2a/B2b of C21, B3I/B2a/B2b of C24 and B2b of C28 frequency bands, indicating that the satellite-induced elevation‑dependent code bias may also appear in different antennas or receivers. However, no similar variations have been found in the existing research with different antennas or receivers [16,17,18] and the systematic variations are observed on the MP combination of B1C due to the signal processing of the receiver [16]. Therefore, more different antennas or receivers are needed to analyze the variation and influence of the BDS-3 satellite-induced code bias.

## 6. Conclusions

The construction of BDS-3 will be completed in an all-round way in 2020. The code bias characteristics for BDS-3 satellites contributes to knowing of the accuracy of satellite navigation signals and further realizes better application. In the paper, we characterize the satellite-induced code bias variation based on the observations of the B1I/B2b/B3I/B1C/B2a frequency bands of the fifteen BDS-3 MEO satellites collected by a 40 m dish antenna. From these validations, the following conclusions were obtained:

(1) The MP combination values of the BDS-3 satellite signals are small and concentrated in a range of about 0.1–0.2 m. The MP combination value differs from each frequency due to signal constructions. The MP combination value of the B1I and B3I frequency band has the largest and smallest RMS value, indicating the weakest and strongest anti-MP ability. In addition, the MP mitigation of the B2a and B2b frequency bands is better than that of the B1I and B1C frequency bands.
(2) The satellite-induced code bias variation of BDS-3 demonstrates different performances on different frequency bands on different satellites, which was greatly reduced compared with that of the BDS-2 satellites. There is obvious elevation‑dependent code bias variation for the C28 B1I/B2b/B3I/B1C/B2a frequency bands, compared with other satellites. Similarly, the MP combination of B3I has an obvious elevation‑dependent variation in a range of 0.1 m for C21/C24/C27/C28/C37. The obvious elevation‑dependent variation of the B2a and B2b frequency bands also exists in most satellites with a range of 0.1 m, but the MP combination values of some satellites are asymmetric with respect to elevation, which is especially obvious for the B1I and BIC frequency bands with elevation‑dependent variations of 0.2 m, which indicates that the code bias variation is not uniquely related to elevation, especially for the B1I/BIC frequency bands.
(3) The magnitude and variation of the MP combination value of each frequency band vary with elevation but do not always increase with a decrease in elevation. The code bias variation with elevations below 30° is the largest and most vulnerable to the interference of MP error, which can be considered in the stochastic model of GNSS data processing. The satellite-induced code variations of two manufacturers of BDS-3 satellites have no obvious differences at elevations larger than 25°.
(4) The code bias of the B1C_data is slightly larger than that of the B1C_pilot, but the difference is small and can be neglected. What’s more, the MP combination time series have similar variations in B1C_data and B1C_pilot.
(5) To explore the performance of the code bias for mass users with normal geodetic antennas, the Xia1 station from iGMAS was selected. Among the frequency bands of the C21/C24/C27/C28/C37 satellites, there still exists elevation‑dependent code bias in the frequency bands B1I/B1C/B3I/B2a/B2b of C21, B3I/B2a/B2b of C24 and B2b of C28 by comparing the MP combination values within the same observation arc, indicating that satellite-induced elevation‑dependent code bias may also appear in different antennas or receivers.

Our conclusions are based on one receiver, however, so they are also worth further verification with other receivers with different configurations. In addition, the influence of the BDS-3 satellite-induced code bias on precision positioning and ambiguity fixing is worth further study using different antennas or receivers.

## Figures and Tables

**Table 1:** Available open service signals transmitted by the new-generation BDS-3 satellites.

Bands	Frequency (MHz)	Modulation Scheme		Code Rate (Mcps)	Compatible with Interoperable Signals
B1I	1561.098	BPSK^1^ (2) QPSK^2^ (10) BPSK (10)		2.046	
B2b	1207.140			10.23	Galileo E5b
B3I	1268.520			10.23	
B1C	1575.420	B1C_pilot	QMBOC^3^ (6, 1)	1.023	GPS L1/Galileo E1
		B1C_data	BOC^4^ (1, 1)		
B2a	1176.450	QPSK (10)		10.23	GPS L5/ Galileo E5a

Abbreviations: ^1^BPSK, binary phase shift keying; ^2^QPSK, quadrature phase shift keying; ^3^QMBOC, quadrature multiplexed binary offset carrier; ^4^BOC, binary offset carrier

**Table 2:** The calculation formula of the MP combination with the available frequency bands.

Frequency Band	Notation	Formula
B1I	M1	$P_{M1}-\frac{f_{M1}^{2}+f_{M5}^{2}}{f_{M1}^{2}-f_{M5}^{2}}L_{M1}+\frac{2f_{M5}^{2}}{f_{M1}^{2}-f_{M5}^{2}}L_{M5}$
B2b	M2	$P_{M2}-\frac{f_{M2}^{2}+f_{M1}^{2}}{f_{M2}^{2}-f_{M1}^{2}}L_{M2}+\frac{2f_{M1}^{2}}{f_{M2}^{2}-f_{M1}^{2}}L_{M1}$
B3I	M3	$P_{M3}-\frac{f_{M3}^{2}+f_{M1}^{2}}{f_{M3}^{2}-f_{M1}^{2}}L_{M3}+\frac{2f_{M1}^{2}}{f_{M3}^{2}-f_{M1}^{2}}L_{M1}$
B1C	M4	$P_{M4}-\frac{f_{M4}^{2}+f_{M3}^{2}}{f_{M4}^{2}-f_{M3}^{2}}L_{M4}+\frac{2f_{M3}^{2}}{f_{M4}^{2}-f_{M3}^{2}}L_{M3}$
B2a	M5	$P_{M5}-\frac{f_{M5}^{2}+f_{M1}^{2}}{f_{M5}^{2}-f_{M1}^{2}}L_{M5}+\frac{2f_{M1}^{2}}{f_{M5}^{2}-f_{M1}^{2}}L_{M1}$

**Table 3:** The RMS values of MP combinations tracked by a 40 m dish antenna with the B1C_data and B1C_pilot signal.

	PRN	RMS (m)							
Signal		C19	C20	C21	C22	C23	C24	C25	C26
B1C_pilot		0.083	0.036	0.046	0.042	0.026	0.058	0.032	0.041
B1C_data		0.085	0.037	0.051	0.045	0.042	0.068	0.045	0.052
	**PRN**	**RMS (m)**							
**Signal**		**C27**	**C28**	**C30**	**C32**	**C33**	**C35**	**C37**	**MEAN**
B1C_pilot		0.043	0.050	0.033	0.035	0.046	0.036	0.066	0.045
B1C_data		0.048	0.060	0.036	0.037	0.054	0.042	0.073	0.052

**Table 4:** The RMS values of MP combinations tracked by two receivers with the 40-m dish antenna and normal geodetic antenna.

PRN	RMS of the Station with the 40 m Dish Antenna (m)					RMS of the Station with the Normal Geodetic Antenna (m)				
	B1I	B2b	B3I	B1C	B2a	B1I	B2b	B3I	B1C	B2a
19	0.13	0.04	0.03	0.06	0.05	0.10	0.10	0.08	0.16	0.11
20	0.07	0.03	0.02	0.03	0.04	0.12	0.13	0.09	0.24	0.13
21	0.07	0.03	0.03	0.05	0.05	0.10	0.11	0.09	0.14	0.11
22	0.07	0.02	0.02	0.03	0.04	0.07	0.09	0.08	0.18	0.09
23	0.04	0.02	0.02	0.03	0.02	0.07	0.08	0.06	0.11	0.08
24	0.06	0.04	0.03	0.06	0.05	0.07	0.10	0.07	0.12	0.09
25	0.05	0.03	0.02	0.03	0.02	0.08	0.09	0.07	0.13	0.10
26	0.06	0.03	0.02	0.03	0.03	0.08	0.10	0.08	0.16	0.11
27	0.05	0.03	0.03	0.04	0.03	0.07	0.09	0.07	0.15	0.09
28	0.05	0.03	0.03	0.05	0.03	0.07	0.10	0.07	0.14	0.09
32	0.04	0.02	0.02	0.03	0.04	0.10	0.09	0.07	0.17	0.09
35	0.04	-	0.02	0.03	0.03	0.06	0.07	0.06	0.12	0.08
37	0.06	0.02	0.03	0.06	0.04	0.08	0.10	0.07	0.14	0.10
MEAN	0.06	0.03	0.02	0.04	0.03	0.08	0.09	0.07	0.15	0.09

## References

[1] Amiri-Simkooei A.R., Jazaeri S., Zangeneh-Nejad F., Asgari J. (2016). Role of stochastic model on GPS integer ambiguity resolution success rate. GPS Solut..

[2] Wanninger L., Beer S. (2015). BeiDou satellite-induced code pseudorange variations: Diagnosis and therapy. GPS Solut..

[3] Xu H., Cui X., Lu M. Satellite-Induced Multipath Analysis on the Cause of BeiDou Code Pseudorange Bias. Proceedings of the China Satellite Navigation Conference (CSNC).

[4] Hauschild A., Montenbruck O., Sleewaegen J.M., Huisman L., Teunissen P.J.G. (2012). Characterization of compass M-1 signals. GPS Solut..

[5] Montenbruck O., Steigenberger P., Prange L., Deng Z., Zhao Q., Perosanz F., Romero I., Noll C., Stürze A., Weber G. (2017). The Multi-GNSS Experiment (MGEX) of the International GNSS Service (IGS)—Achievements, prospects and challenges. Adv. Space Res..

[6] Gisbert J.V.P., Batzilis N., Risueño G.L., Rubio J.A. GNSS Payload and Signal Characterization using a 3m Dish Antenna. Proceedings of the 25th International Technical Meeting of the Satellite Division of The Institute of Navigation (ION GNSS).

[7] Montenbruck O., Rizos C., Weber R., Weber G., Neilan R., Hugentobler U. (2013). Getting a grip on multi-GNSS—The international GNSS service MGEX campaign. GPS World.

[8] Li M., Qu L., Zhao Q., Guo J., Su X., Li X. (2014). Precise Point Positioning with the BeiDou Navigation Satellite System. Sensors.

[9] Jiang W., Zhao W., Chen H., Liu X., An X., Chen Q. (2019). Analysis of BDS Fractional Cycle Biases and PPP Ambiguity Resolution. Sensors.

[10] Li P., Zhang X., Guo F. (2016). Ambiguity resolved precise point positioning with GPS and BeiDou. J. Geod..

[11] Lei W., Wu G., Tao X., Bian L., Wang X. (2017). BDS satellite induced code multipath: Mitigation and assessment in new-generation IOV satellites. Adv. Space Res..

[12] Xie X., Geng T., Zhao Q., Liu J., Wang B. (2017). Performance of BDS-3: Measurement quality analysis, precise orbit and clock determination. Sensors.

[13] Zhang X., Li X., Lu C., Wu M., Pan L. (2017). A comprehensive analysis of satellite-induced code bias for BDS-3 satellites and signals. Adv. Space Res..

[14] Zhang X., Wu M., Liu W., Li X., Yu S., Lu C., Wickert J. (2017). Initial assessment of the COMPASS/BeiDou-3: New-generation navigation signals. J. Geod..

[15] Zhou R., Hu Z., Zhao Q., Li P., Wang W., He C., Cai C., Pan Z. (2018). Elevation-dependent pseudorange variation characteristics analysis for the new-generation BeiDou satellite navigation system. GPS Solut..

[16] Zhang Z., Li B., Nie L., Wei C., Jia S., Jiang S. (2019). Initial assessment of BeiDou‑3 global navigation satellite system: Signal quality, RTK and PPP. GPS Solut..

[17] Xie X., Fang R., Geng T., Wang G., Zhao Q., Liu J. (2018). Characterization of gnss signals tracked by the igmas network considering recent bds-3 satellites. Remote Sens..

[18] Zhang B., Jia X., Sun F., Xiao K., Dai H. (2019). Performance of BeiDou-3 Satellites: Signal Quality Analysis and Precise Orbit Determination. Adv. Space Res..

[19] CSNO (2016). BeiDou Navigation Satellite System Signal in Space Interface Control Document—Open Service Signal.

[20] CSNO (2018). Development of the BeiDou Navigation Satellite System.

[21] CSNO (2019). Development of the BeiDou Navigation Satellite System.

[22] Xiao W., Liu W., Sun G. (2016). Modernization milestone: BeiDou M2-S initial signal analysis. GPS Solut..

[23] Yang Y., Gao W., Guo S., Mao Y., Yang Y. (2019). Introduction to BeiDou-3 navigation satellite system. Navigation.

[24] Leick A., Rapoport L., Tatarnikov D. (2015). GPS Satellite Surveying.

[25] Defraigne P., Bruyninx C. (2007). On the link between GPS pseudorange noise and day-boundary discontinuities in geodetic time transfer solutions. GPS Solut..

[26] Estey L.H., Meertens C.M. (1999). Teqc: the multi-purpose toolkit for gps/glonass data. GPS Solut..

[27] Silva P.F., Silva J.S., Peres T.R. Galileo AltBOC signal processing for precise positioning Experimental results. Proceedings of the 25th International Technical Meeting of the Satellite Division of the Institute of Navigation (ION GNSS).

[28] Zhao Q., Wang C., Guo J., Wang B., Liu J. (2017). Precise orbit and clock determination for beidou-3 experimental satellites with yaw attitude analysis. GPS Solut..

